# "What is the best method of family planning for me?": a text mining analysis of messages between users and agents of a digital health service in Kenya

**DOI:** 10.12688/gatesopenres.12999.1

**Published:** 2019-05-29

**Authors:** Eric P Green, Alexandra Whitcomb, Cynthia Kahumbura, Joseph G Rosen, Siddhartha Goyal, Daphine Achieng, Ben Bellows

**Affiliations:** 1 Duke Global Health Institute, Duke University, Durham, NC, 27708, USA; 2 Nivi Inc., 40 Tall Pine Drive, Sudbury, MA, 01776, USA; 3 AskNivi Limited, Windsor House, University Way, Nairobi, PO Box 34430-00100, Kenya; 4 Population Council, Plot #3670, Mwaleshi Rd, Lusaka, 10101, Zambia; 5 Population Council, 4301 Connecticut Ave NW # 280, Washington, DC, 20008, USA

**Keywords:** digital health, reproductive health, sms, text mining, kenya

## Abstract

**Background**: Text message-based interventions have been shown to have consistently positive effects on health improvement and behavior change. Some studies suggest that personalization, tailoring, and interactivity can increase efficacy. With the rise in artificial intelligence and its incorporation into interventions, there is an opportunity to rethink how these characteristics are designed for greater effect. A key step in this process is to better understand how users engage with interventions. In this paper, we apply a text mining approach to characterize the ways that Kenyan men and women communicated with the first iterations of *askNivi*, a free sexual and reproductive health information service.

**Methods**: We tokenized and processed more than 179,000 anonymized messages that users exchanged with live agents, enabling us to count word frequency overall, by sex, and by age/sex cohorts. We also conducted two manual coding exercises: (1) We manually classified the intent of 3,834 user messages in a training dataset; and (2) We manually coded all conversations between a random subset of 100 users who engaged in extended chats.

**Results**: Between September 2017 and January 2019, 28,021 users (mean age 22.5 years, 63% female) sent 87,180 messages to *askNivi,* and 18 agents sent 92,429 replies. Users wrote most often about family planning methods, contraception, side effects, pregnancy, menstruation, and sex, but we observed different patterns by sex and age. User intents largely reflected the marketing focus on reproductive health, but other topics emerged. Most users sought factual information, but requests for advice and symptom reports were common.

**Conclusions**: Young people in Kenya have a great desire for accurate and reliable information on health and wellbeing, which is easy to access and trustworthy. Text mining is one way to better understand how users engage with interventions like *askNivi* and maximize what artificial intelligence has to offer.

## Introduction

An active area of research and practice falling under the umbrella of digital health is the development and evaluation of mobile text message-based interventions for health improvement and behavior change. Health topics and behaviors targeted in these interventions have included disease management, medication adherence, smoking cessation, weight loss, sexual health, contraception, among many others ( Hall *et al*., 2015). Until recently, the most common channel of communication in these interventions has been short message service, better known as SMS or text messaging, a feature available on all mobile phones, which lets users read and compose alphanumeric messages of up to 160 characters. Since at least 2015, however, mobile phone messaging applications such as WhatsApp and Facebook Messenger have eclipsed SMS in terms of daily message volume ( Perez, 2016; Sparkes, 2015).

Research findings pointing to the efficacy of text message-based interventions have been summarized in more than a dozen systematic reviews and meta-analyses, as well as several systematic reviews of reviews ( Hall *et al*., 2015; Househ, 2016). Meta-analyses have consistently reported average standardized effect sizes of 0.24 (0.16–0.32; Armanasco *et al*., 2017), 0.29 (0.22–0.36; Orr & King, 2015), and 0.33 (0.24–0.39; Head *et al*., 2013) across prevention and health promotion interventions targeting diverse health topics. Given the low unit cost of communication via text message, these modest effect sizes suggest that text message-based interventions have the potential to be highly cost-effective.

While the evidence is mixed ( Armanasco *et al*., 2017), several reviews report that intervention personalization (e.g., including a user’s name), tailoring (e.g., outbound messages are determined by a user’s previous responses), and interactivity (e.g., two-way vs one-way messaging) may boost the efficacy of these interventions ( Hall *et al*., 2015). It is important to consider, however, that these findings represent an initial signal from *first generation* text message-based interventions. A *second generation* of interventions is emerging with the rapid integration of artificial intelligence (AI) into health applications, which could enable greater personalization, smarter tailoring, and more engaging interactivity ( Shaban-Nejad *et al*., 2018; USAID, 2019; Wahl *et al*., 2018).

A well-known challenge in creating AI-informed applications for health is the need for large amounts of training data. A related challenge is that the training data must be relevant to the context of the intended end users. For instance, systems trained on data from US-based patients may not generalize to the needs of users living in low- and middle-income countries ( USAID, 2019). To create this new generation of applications that will benefit diverse populations, we should apply design thinking principles ( Brown, 2008) and seek to understand how users engage with our interventions.

One approach for exploring and understanding user engagement is text mining. Text mining is the practice of using automated tools to examine large amounts of free-form text, summarize the contents, uncover interesting patterns, and generate new insights from the data. The most active areas of research in text mining for health have been the analysis of electronic medical records ( Koleck *et al*., 2019; Kreimeyer *et al*., 2017) and social media messages ( Sinnenberg *et al*., 2017). There have been few published analyses of two-way communication transcripts. Ye *et al*. (2010) published a systematic review of studies, which examined e-mail communication between patients and providers. Blanc *et al*. (2016) conducted a content analysis of messages that users in Nigeria sent to a sexual and reproductive health question and answer service called *MyQuestion*. Despite a large and growing body of evidence around the efficacy of text message-based interventions, there has been scant attention to the study of user engagement through text mining.

In this paper, we conduct a text mining analysis of the inbound and outbound messages to *askNivi*, a free sexual and reproductive health information service currently operating in Kenya and India ( Nivi, 2018). Users can send free-form messages to *askNivi* via SMS or Facebook Messenger and interact with automated conversation modules or live customer success agents. The aim of *askNivi* is to provide health information, referrals to health products and services, and encouragement to take action that will promote health and well-being. The objective of this analysis is to characterize the ways that Kenyan men and women communicated with the first iterations of *askNivi* about their health inquiries to inform future content development, tailoring, and automation.

## Methods

The data for this secondary analysis comes from a query of the Kenya *askNivi* database for all valid inbound and outbound SMS messages handled by customer success agents between September 2017 and January 2019. The query resulted in 179,609 total messages (87,180 inbound and 92,429 outbound). The data were anonymized prior to analysis.

### Language detection

We conducted all data processing and analysis in R version 3.5 ( R Core Team, 2018). To detect the language of each message, we used the cld2 package (v1.2; Ooms, 2018a) to access Google’s Compact Language Detector 2 ( Sites, 2013), a naïve Bayes classifier that probabilistically detects 83 languages, including English and Swahili. The classifier detected that users sent 47% of messages in English, 25% in Swahili, and failed to detect either language in 28% of incoming messages. When the language could not be automatically detected for a message, we set the missing language label to the dominant language detected in the user’s inbound messages during the same calendar week. For instance, if a person sent 10 messages in one week (e.g., six English, two Swahili, and two with no language detected), we set the two missing language labels to English.

### Text mining

We used the tidytext package (v0.2.0; Silge & Robinson, 2016) to analyze word frequency and relationships in all inbound messages. The first step was to tokenize each message into its component words, strip all punctuation, and convert the tokens to lowercase. We filtered out 1149 English stop words (e.g., "a", "the", "and") from three lexicons compiled in the tidytext package and 74 Swahili stop words from a multi-language collection of stop words ( Diaz, 2017). We added custom stop words to these lists for both languages based on our initial review of the tokens. See *Extended data* for a full list ( Green, 2019).

Prior to counting the frequency of individual word tokens, we also tokenized each message by consecutive words to identify the most common bigrams. This allowed us to tally key terms as pairs rather than as individual words. For instance, when the word “family” immediately preceded the word “planning”, we tallied “family” as part of the bigram “family planning”. However, if “family” occurred on its own, e.g., “I do not want to start a family”, then we tallied family as an individual term.

We used the hunspell package (v3.0; Ooms, 2018b) to detect possible misspellings and suggest corrections but ultimately decided to only accept suggestions for English words that appeared fewer than four times in the corpus, given our concerns about reliability. We did not accept any Swahili spelling suggestions.

We used the textstem package (v0.1.4; Rinker, 2018) to conduct lemmatization on the English words and identify the base form of each word - its lemma. We opted to conduct lemmatization over stemming to avoid the creation of non-word stems. Following this process, we ran an initial word frequency count and conducted a manual review to identify synonyms that could be combined into one label (e.g., “period” and “menses” combined into “period”; see *Extended data* for the full list; Green, 2019). After combining like terms, we conducted another frequency analysis using the tidytext package to get the final count of each word or key bigram in the corpus of messages.

### Intent analysis

When a user sends a question or statement to *askNivi*, a natural language processing algorithm trained on past submissions classifies the user’s intent. For instance, a user might send a message that reads, “I want to find a good method of family planning”. The intent behind this question - what the user wants to know or do - is “find a method of contraception”. To develop the training set to build a predictive model for automated intent classification, we built a simple web application that enabled our agents to read each question and manually label the intent ( Green, 2018). Each question was presented for classification until two different agents agreed on the best intent label. Through this process, we manually classified 3,303 English and Swahili language messages in the training dataset. In this paper, we present a descriptive summary of user intent.

### Conversation analysis

To analyze the structure of extended exchanges between users and agents, we selected a random sample of 50 men and 50 women who sent at least seven English messages to *askNivi* during one calendar week. A member of the team read the collection of 2,590 messages and qualitatively coded the number of distinct conversations each user had with agents, the topics discussed in each conversation, and the message-level components of each exchange (e.g., questions, responses, greetings).

### Preparation of data for sharing

Anonymized message meta-data with calendar week time stamps is archived along with the tokenized word frequencies ( Green, 2019). To prevent accidental sharing of private information due to possible imperfect anonymization, we omitted terms that appear fewer than three times in the corpus.

### Ethical statement

Our study protocol was screened by the Duke University Institutional Review Board and determined to be exempt from further review.

## Results

### Descriptive summary of *askNivi* usage

***Users.*** Between week 38 of 2017 and week 5 of 2019, 28,021 users sent 87,180 messages to *askNivi,* and 18 agents sent 92,429 replies ( Green, 2019). Questions that required input from a medical advisor were handled by nurses at a local maternity hospital. Nurse replies accounted for 1.4% of these outbound messages. Table 1 displays user characteristics. Nearly two-thirds of users were female, and roughly half indicated they preferred to receive messages in English. The average user was 22.5 years old (SD=6.4).

**Table 1.** Characteristics of users.

Variable	Values	Missing
Users, N	28,021	0
Female, %	63.2	12,528
Prefers English, %	46.7	303
Mean Age (SD)	22.5 (6.4)	13,801

*Note.* User sex was not routinely captured during the entire period covered by the dataset.

SD, standard deviation

***Message-level language.*** Of the 87,180 inbound messages received by *askNivi*, 63% were written in English, 36% in Swahili, and 2% messages could not be labeled after imputing the dominant language. Nearly one out of five users (18%) sent at least one message in a language that did not match their stated language preference, and 14% of all inbound messages were discordant in terms of a user’s stated language preference and the detected language.

**Figure 1.**
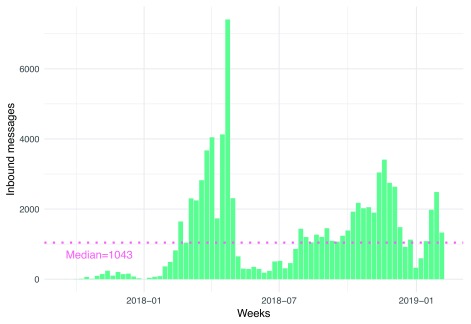
Volume of inbound messages per week.

***Patterns of engagement.*** The median user sent 2.0 messages during the period covered by the dataset (M=3.1, SD=4.3). Inbound message volume fluctuated over time. Figure 1 shows a spike in total inbound message volume during the middle of 2018, followed by a drop-off that corresponded to an intentional pause in marketing of the service. *askNivi* ended the study period with roughly 2000 inbound messages per week.

**Figure 2.**
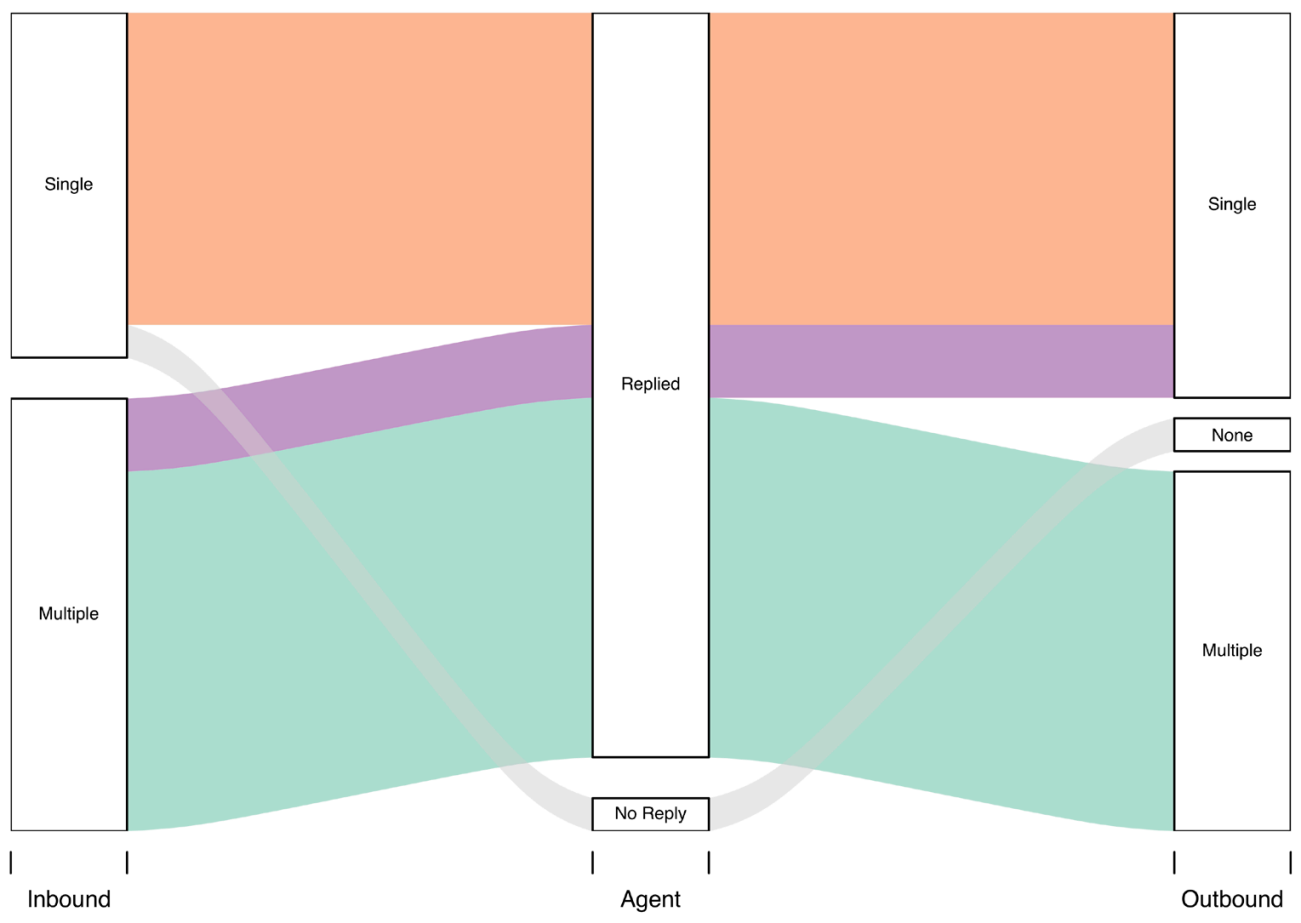
Alluvial diagram of inbound and outbound messages.

Figure 2 shows three main patterns in communication: 40% of users sent one message and received one reply (orange), 9% sent multiple messages and received one reply (purple), and 46% sent and received multiple messages (green). This pattern was broadly similar for women and men, but men were 1.7 times as likely as women to send only one message to *askNivi*.

### Text analysis

**Figure 3.**
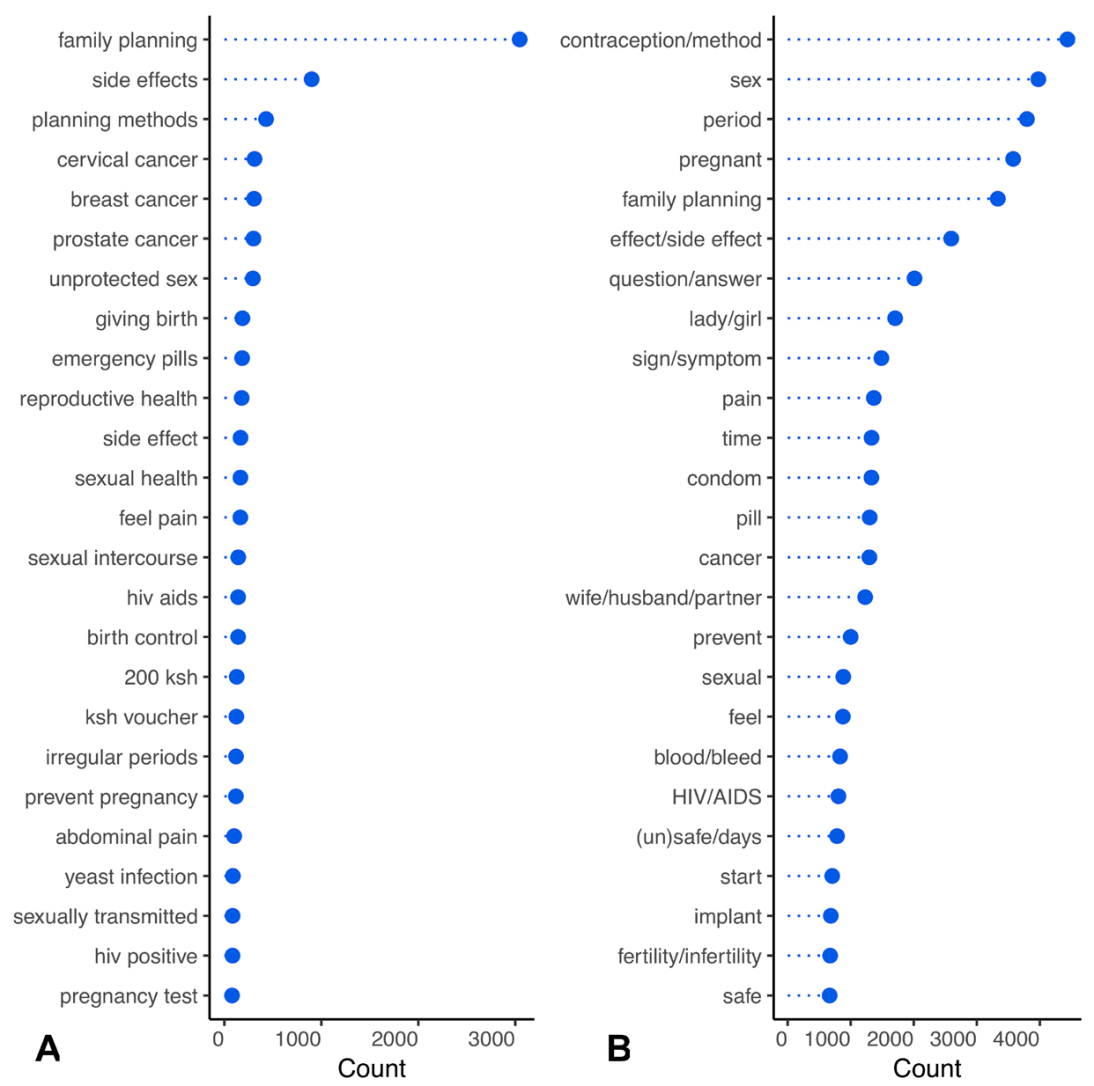
Most frequently occurring bigrams ( **A**) and words ( **B**) in incoming messages (English). ksh, Kenyan shillings; HIV, human immunodeficiency virus; AIDS, acquired immunodeficiency syndrome.

Figure 3 shows the top 25 most frequently occurring pairs of adjacent words, or bigrams (A), and single words (B) in English language messages sent to *askNivi*. Users wrote most often about family planning methods, contraception, side effects, pregnancy, menstruation, and sex.

**Figure 4.**
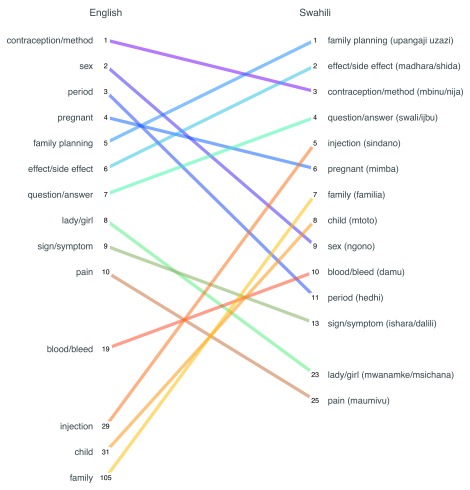
Rank order of the most common words used in incoming messages by language. This is a forced-rank chart because two words could have the same frequency of usage, but no ties are awarded.

As Figure 4 shows, the most frequently used terms are broadly similar between English and Swahili messages. Eight of the top 10 English terms fell within the Swahili top 15 ranking.

**Figure 5.**
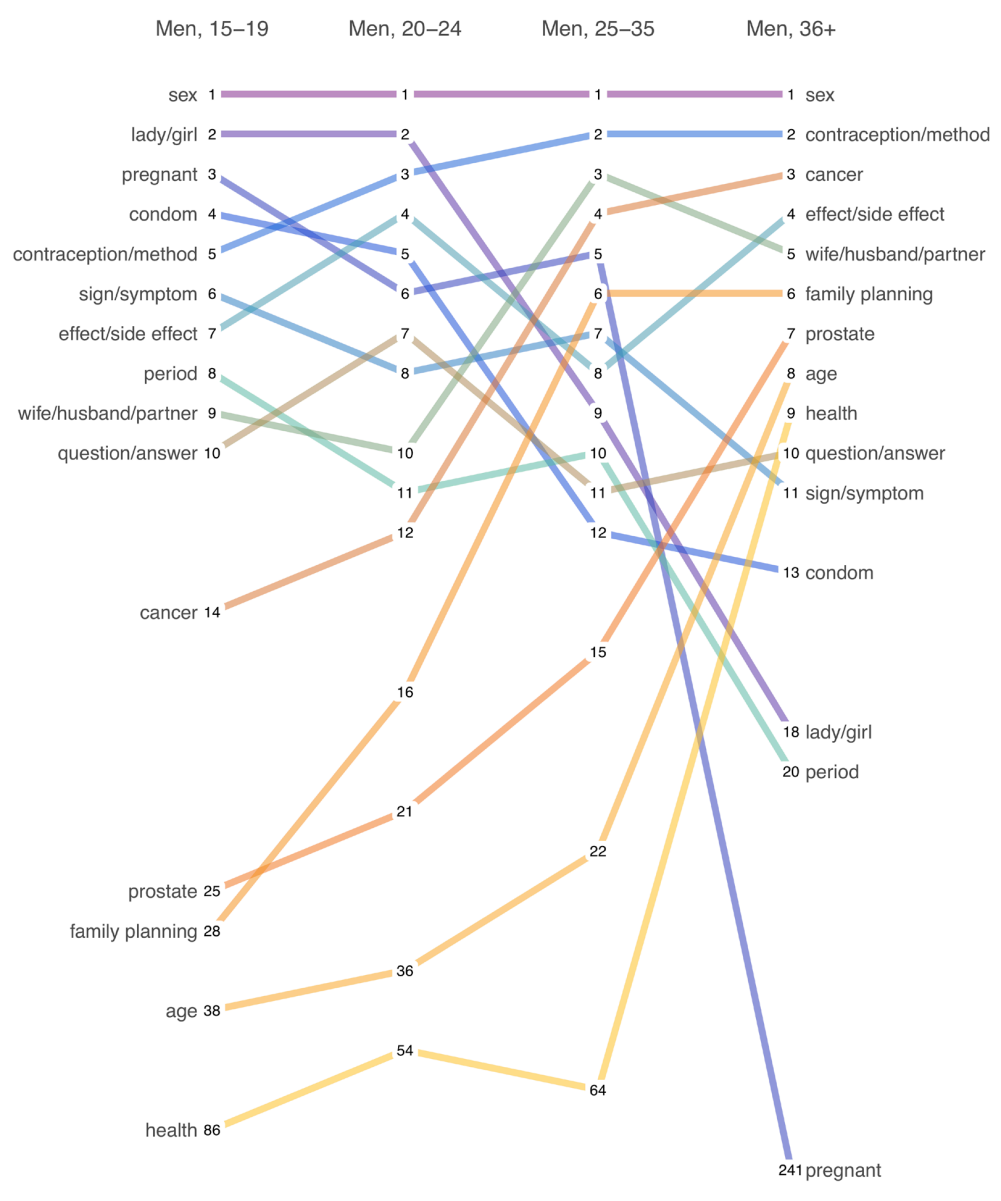
Rank order of the most common words used in incoming messages (English) by age category, men. This is a forced-rank chart because two words could have the same frequency of usage, but no ties are awarded.

**Figure 6.**
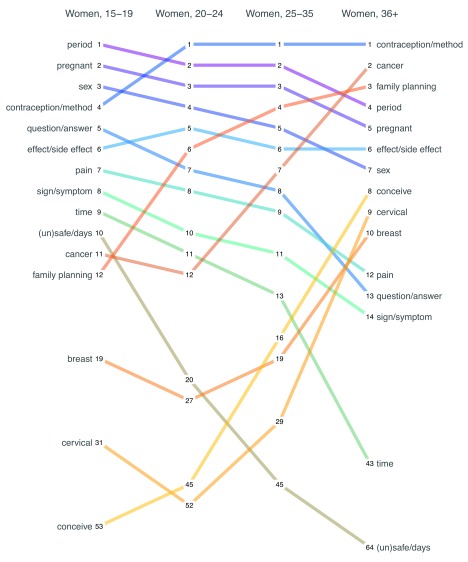
Rank order of the most common words used in incoming messages (English) by age category, women. This is a forced-rank chart because two words could have the same frequency of usage, but no ties are awarded.

It is informative to examine differences in word frequency by sex and age. Figure 5 presents age disaggregated data for men, and Figure 6 presents age disaggregated data for women. An easy way to read these plots is to start at the top left, which represents the most frequent word used by adolescents.

In the case of Figure 5, the most frequent English word used by adolescent boys was sex. Follow this line across to see that it remains the most frequent word used by men of all age categories. The same is not true for the word pregnant. ‘Pregnant’ was the third most common word used by adolescent boys (as in preventing pregnancy), but it fell to eighth among men in their 20’s and mid-30’s, and all the way to a rank of 241 among older men. The chart can also be read from right to left starting with the most frequent terms used by older men and tracing back through younger age groups. Doing so reveals that cancer and prostate were frequent topics for older men, but less so among younger men.

Figure 6 presents the same ranking of English word frequency for female age groups. The terms ‘period’ and ‘pregnant’ were common topics across all female age categories. However, young women were much more likely to write about how to identify their safe and unsafe days compared to older women. Conversely, older women chatted more frequently about family planning, conception, and cancers of the breast and cervix. Compared to men, women chatted less about sex overall, and the frequency declined with age.

**Figure 7.**
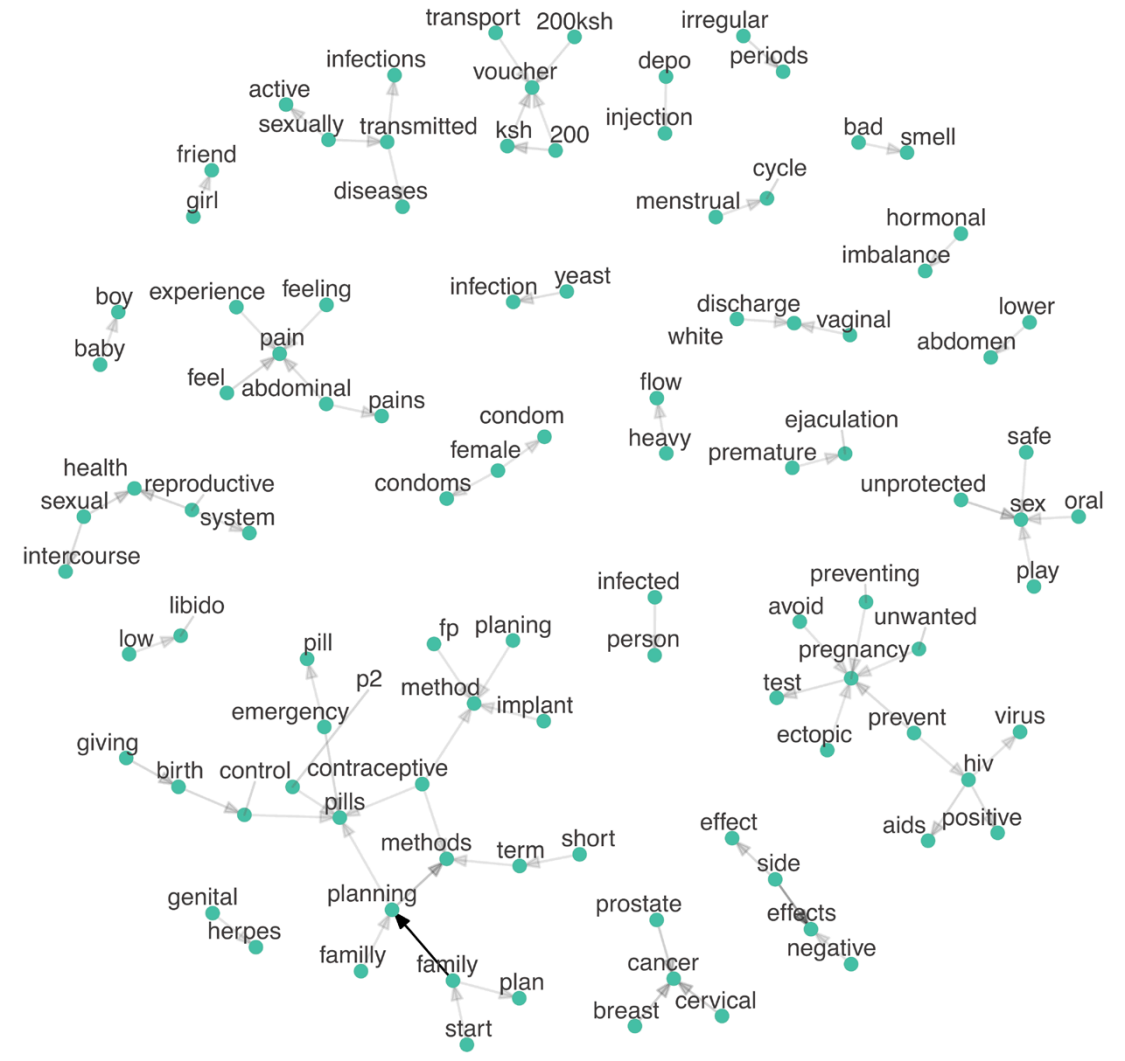
Network graph of most frequent English bigrams. The points (nodes) represent words, the lines (edges) represent the most frequent connections between words, and the arrows indicate the temporal ordering of the words. KSH, Kenyan Shillings; p2, Postinor-2; HIV, human immunodeficiency virus; AIDS, acquired immunodeficiency syndrome; FP, family planning

Figure 7 visualizes common English bigrams as a network graph. The points (nodes) represent words, the lines (edges) represent the most frequent connections between words, and the arrows indicate the temporal ordering of the words. For instance, several words (‘unprotected’, ‘safe’, ‘oral’, and ‘play’), point to the word ‘sex’, reflecting the different ways that users chatted about sex. The word ‘prevent’ bridges two topic clusters that people want to avoid: pregnancy and HIV. For instance, users asked, “How can i prevent unwanted pregnancies?” and “Is the use of condoms during sex completely prevent any HIV infection??”.

**Figure 8.**
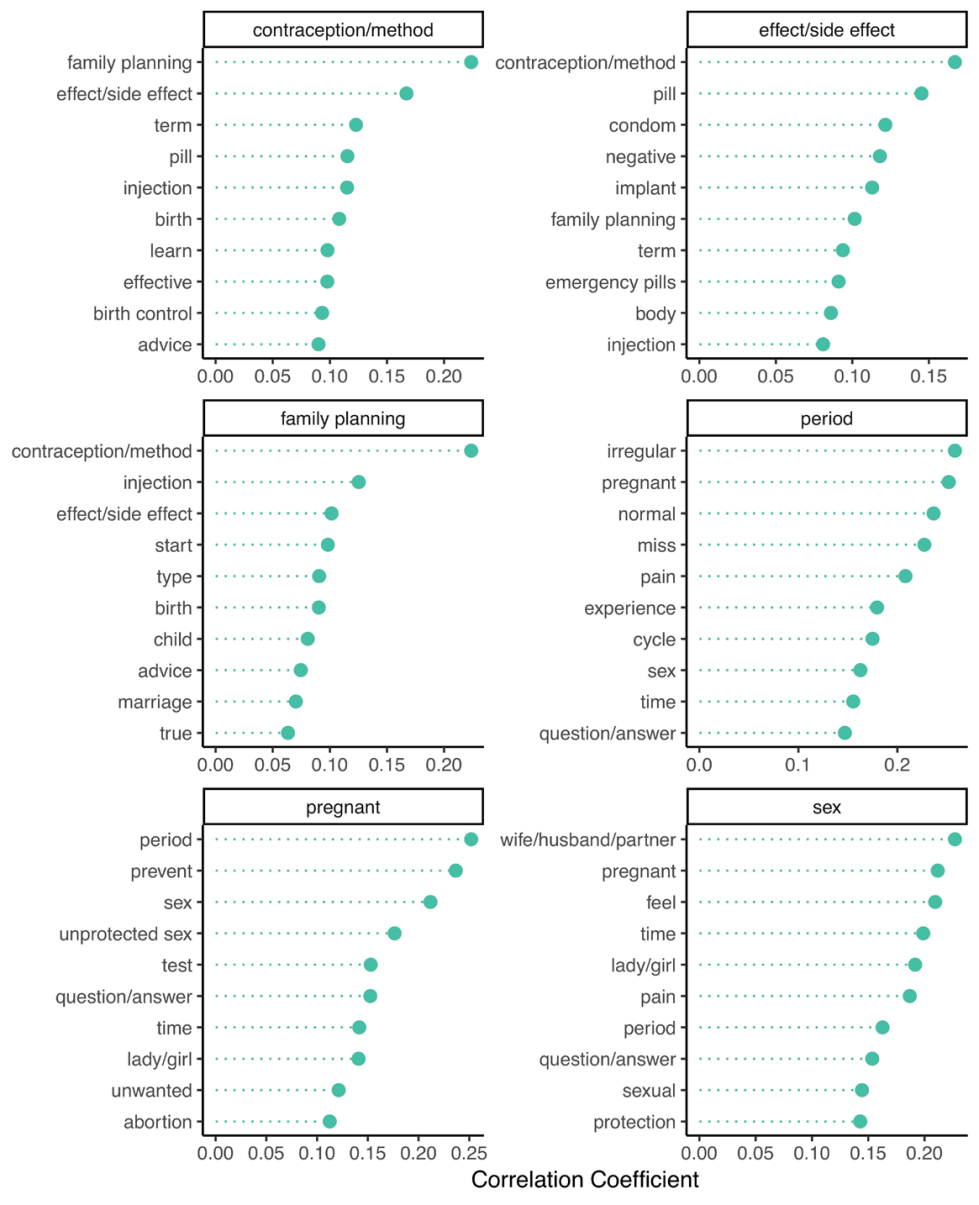
Top pairwise correlations (phi) between English words.

In addition to exploring the relationships between pairs of adjacent words, we also examine how English words co-occur in conversations, regardless of their position in individual messages. Figure 8 shows the words that are most associated with several key terms such as 'contraception' and 'period'. For instance, when asking about contraception and method options, users often want to know about side effects and to learn which methods are effective. Conversations about periods often involve descriptive words like 'irregular', 'normal', and 'pain', and include questions about the possibility of pregnancy with missed periods. For instance, users asked questions like, “Why are my periods irregular?” and “What are some ways that can reduce abdominal pains during menstrual periods?”.

### Intent analysis

**Figure 9.**
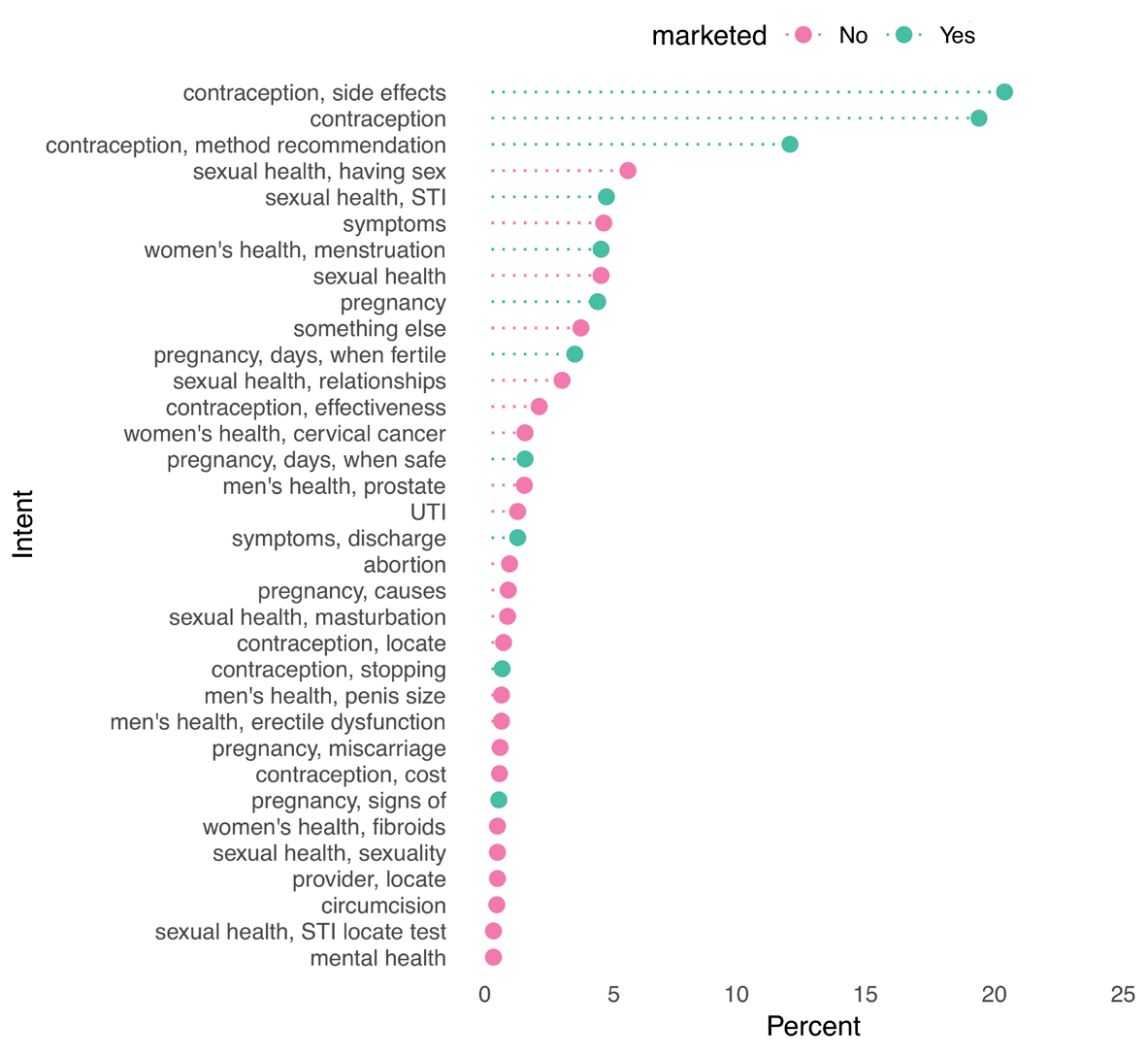
Distribution of classified intents by marketing history. STI, sexually transmitted infection; UTI, urinary tract infection.

In order to build a training dataset that would enable automated classification of user intent, we manually classified a subset of 3,834 initial utterances from users (English and Swahili). Figure 9 displays the distribution of intents and indicates whether the intent was part of an *askNivi* marketing campaign. This figure shows that the most frequent intents were related to contraception, a major focus of *askNivi* marketing. It also shows, however, that users asked questions on topics that *askNivi* was not marketing at the time, including sexually transmitted infections, symptoms, sexual health, and relationships.

**Figure 10.**
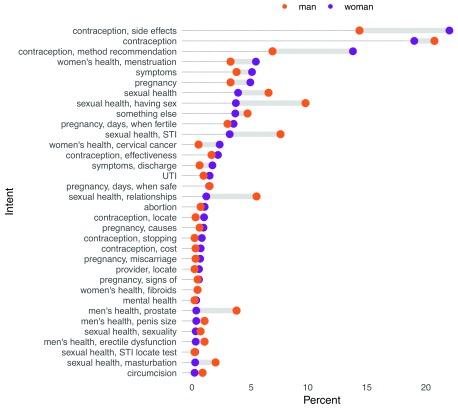
Distribution of classified intents by gender. STI, sexually transmitted infection; UTI, urinary tract infection.

*askNivi* was initially marketed to adolescent girls and young women, but a high demand among men led to an expansion of the intended market audience. Figure 10 shows the distribution of user intent by sex. Compared to women, men were more interested in questions related to sexual health, relationships, and sexually transmitted infections. However, overall, contraception was still the dominant theme among men and women.

### Conversation analysis

We manually coded 2,590 English language messages exchanged between *askNivi* agents and 100 users (50 men, 50 women), selected at random from the pool of high-engagement users. The average age of this subset of users was 21.4 years (SD=3.7). Table 2 summarizes key characteristics of these conversations.

**Table 2.** Characteristics of conversations.

Group	*N*	Messages	Conversations	Convos/person Mean (SD)	Messages/convo Mean (SD)	Topics/convo Mean (SD)
All	100	2590	207	2.1 (1.8)	12.5 (8.9)	2.6 (1.8)
Men	50	1276	101	2.0 (1.5)	12.6 (8.8)	2.8 (1.9)
Men, 15–19	10	271	18	1.8 (1.6)	15.1 (9.6)	3.1 (2.1)
Men, 20–24	29	699	57	2.0 (1.5)	12.3 (9.0)	2.6 (1.9)
Men, 25–35	8	259	22	2.8 (1.7)	11.8 (8.3)	3.1 (2.1)
Women	50	1314	106	2.1 (2.0)	12.4 (9.1)	2.3 (1.5)
Women, 15–19	21	579	51	2.4 (2.7)	11.4 (8.2)	2.3 (1.5)
Women, 20–24	19	506	41	2.2 (1.4)	12.3 (9.0)	2.4 (1.8)
Women, 25–35	7	181	10	1.4 (0.8)	18.1 (13.5)	2.6 (1.0)

*Note*. Sex x Age *N*'s do not sum to 50 because of missing age values.

Convos/person, conversations per person; messages/convo, messages per conversation; topics/convo, topics per conversation; SD, standard deviation

We determined that these 100 users engaged in a total of 207 distinct conversations, for an average of 2.1 conversations per person (SD=2.1, median=1.0). On average, conversations consisted of 12.5 messages (SD=8.9): 6.6 messages sent by users and 5.9 replies sent by agents. 72% of user messages came in the form of questions or requests. We classified these questions as shown in Table 3. Most user questions sought factual information, such as requests to define or explain concepts and questions about causes (60.1%). Among the 516 requests for information, 4.7% asked about common myths (e.g., "Is it true that if one have a kiss with someone positive, the are high chances of being affected?”).

**Table 3.** Distribution of user questions/requests.

Category	Example	Percent
1. Requests for factual information about causes	Can someone using an implant conceive immediately?	48.9
2. Requests for factual information about the meaning of concepts/terms	What is family planning?	11.2
3. Requests for advice	What are the best contraceptive options for avoiding pregnancy?	26.0
4. Questions about access to services and products	Which facility should I visit please?	1.5
5. Reporting symptoms, requesting diagnosis	I gave birth 1year ago from then I have never seen my periods am I safe or I have a problem?	10.6
6. Other		1.9

**Figure 11.**
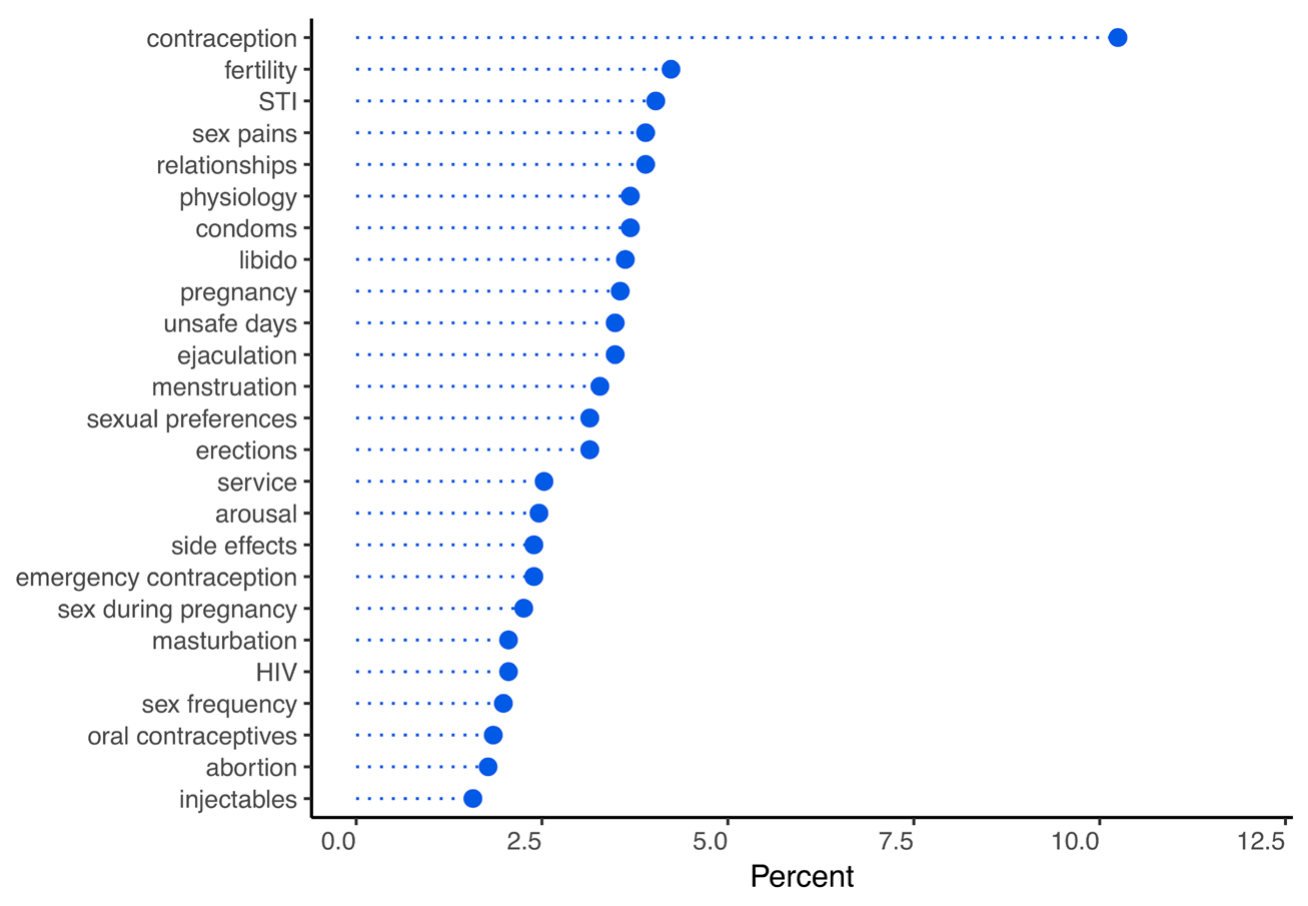
Distribution of topics that appeared most frequently in multiple-topic conversations, top 25 topics shown. STI, sexually transmitted infection; HIV, human immunodeficiency virus.

Over half (128) of the 207 conversations involved multiple topics. In the average conversation, users and agents discussed 2.6 topics ( *SD*=1.8). As shown in Figure 11, the topics that appeared most frequently in multiple-topic conversations were contraception, fertility, sexually transmitted infection (STI), relationships, and sex pains. For instance, multiple-topics conversations about contraception were most frequently paired with discussions of unsafe days, menstruation, and emergency contraception.

## Discussion

This paper presents the results of a text mining analysis of 179,609 SMS messages, which users exchanged with customer success agents of *askNivi*, a free digital health service operating in Kenya and India. First and foremost, our descriptive findings from several initial iterations of the Kenya *askNivi* service are useful internally as we seek to improve and expand the product. In particular, content analysis is informing the development of automated conversations and the creation of an ontology that links user intents and topics to a cascade of conversations, thereby allowing us to further tailor the content of *askNivi* and make automated, personalized recommendations.

Externally, this paper makes two contributions. First, the methodology presented here and documented in the data and code repository ( Green, 2019) offers researchers and practitioners a reproducible example of how to apply text mining techniques to text message-based interventions. We have not encountered any similar examples in the evaluation literature that is largely focused on issues of feasibility, acceptability, and efficacy. Certainly, the techniques presented here could be useful for all three of these aims. Second, the analysis adds to our understanding of how our Kenyan users, in particular adolescents and young adults (mean age of 22.5 years), converse on private networks about topics related to sexual and reproductive health.

We can compare our results directly to a comprehensive qualitative analysis of text messages sent to a question and answer service in Nigeria called *MyQuestion* ( Blanc *et al*., 2016). The most common topics on both platforms followed the dominant marketing focus. Blanc *et al*. (2016) reported that *MyQuestion* began as a platform for HIV/AIDS information; *askNivi* was originally developed to help women learn about and access family planning. Both services also observed a substantial volume of messages about topics not marketed, such as health symptoms and diseases like cancer.

The majority of messages sent to both services took the form of questions seeking factual information about the meaning of health concepts and causes of health conditions. In the case of *askNivi*, many users wanted to talk about different aspects of contraception, from how to find the best method to effectiveness and side effects. We found that nearly all messages not requesting information could be grouped into Blanc *et al.*’s classification scheme of requests for advice, questions about access to services and products, and reports of symptoms/requests for a diagnosis.

This paper adds to the *MyQuestion* analysis by Blanc *et al*. (2016) because the anonymized *askNivi* messages were linked to data on users’ sex and age, allowing us to disaggregate the results. Doing so revealed interesting differences across both demographic dimensions. For instance, young people chatted more about how to avoid pregnancy and practice safe sex, whereas older users asked more about symptoms and health issues like cancer. Men more often wanted information and advice on sexual health and relationships, while women more commonly sought contraception recommendations. These insights can be used to create targeted marketing campaigns and to develop content that will increase user engagement.

Although we demonstrate that users who communicated in Swahili chatted about very similar topics compared to English users, a limitation of our paper is that we did not fully replicate each analysis for the Swahili corpus of messages. This is because we found that existing open source text mining tools for Swahili are less developed compared to the English-language tools. Another limitation of this study is that we may not have a representative sample of Kenyan users overall or by demographic group. While the service was free to use, mobile phone access is nearly universal in Kenya, and marketing efforts included online digital marketing and offline community mobilization, *askNivi* users are likely to be more educated on average compared to the Kenyan population. Furthermore, nearly two-thirds of users were female, following the initial marketing of the service. The men who took the initiative to contact *askNivi* might be different from the general population of potential male users in unmeasured ways. Despite these limitations, the similarities observed with the *MyQuestion* analysis in Nigeria is modest evidence for generalizability in an Anglophone African context.

## Conclusions

The early *askNivi* experience demonstrates that young people in Kenya have a great need for accurate and reliable information on health and wellbeing that is easy to access and trustworthy, replicating what has been observed in other contexts like Nigeria ( Blanc *et al*., 2016). As services like *askNivi* and *MyQuestion* increase in popularity, users go beyond the marketed offering to reveal unmet needs for information, recommendations, and referrals. Text mining is a relatively simple approach for exploring these trends and probing how user needs and interests differ across groups like age cohorts and sex. As artificial intelligence is increasingly incorporated into text message-based interventions like *askNivi*, the opportunities for intervention personalization, tailoring, and interaction will grow. Text mining is one way to better understand how users engage with these interventions and maximize what artificial intelligence has to offer.

## Data availability

### Underlying data

Anonymized message meta-data with calendar week time stamps is archived along with the tokenized word frequencies. Raw message content is not available due to privacy concerns and limitations on user information that cannot be shared publicly. However, the data repository includes (1) a tutorial with a sample of anonymized raw data to demonstrate how to conduct the same analysis on new data; and (2) anonymized message meta-data and tokenized word frequencies to reproduce the analysis, figures, and tables presented in this manuscript.

Zenodo: ericpgreen/asknivi-text-mining-2019: zenodo. https://doi.org/10.5281/zenodo.2653865 ( Green, 2019)

This project contains the following underlying data within the ‘ericpgreen-asknivi-text-mining-2019-v1-2\input’ folder:

- convos_coded.csv (conversation data)
- df_tok_en_wordUser.csv (words by user)
- intents.csv (intent classifications)
- marketed.csv (counts of marketed intents)
- metadata.csv (meta data about messages)
- tok_bi_en.csv (bigram counts, English)
- tok_en.csv (single word frequency, English)
- tok_en_f.csv (single word frequency, females by age category, English)
- tok_en_g.csv (single word frequency, by gender, English)
- tok_en_m.csv (single word frequency, males by age category, English)
- tok_sw.csv (single word frequency, Swahili)

### Extended data

Zenodo: ericpgreen/asknivi-text-mining-2019: zenodo. https://doi.org/10.5281/zenodo.2653865 ( Green, 2019)

This project contains the following extended data within the ‘ericpgreen-asknivi-text-mining-2019-v1-2’ folder:

- README.md (list of all R packages, instructions required to reproduce the analysis, and a tutorial for running the message tokenization on a sample of raw data)
- manuscript.Rmd (text and code to reproduce the analysis and manuscript)

This project contains the following extended data within the ‘ericpgreen-asknivi-text-mining-2019-v1-2\input\example’ folder:

- modifications-en.csv (custom modifications to relabel words and collapse synonyms)
- stop.csv (custom stop words)

Data are available under the terms of the Creative Commons Attribution 4.0 International license (CC-BY 4.0).

### Software availability

Source code available from: https://github.com/ericpgreen/asknivi-text-mining-2019

Archived source code at time of publication: https://doi.org/10.5281/zenodo.2653865 ( Green, 2019)

License: Creative Commons Attribution 4.0 International license (CC-BY-4.0)
