# Supplementary material for: "What is the best method of family planning for me?": a text mining analysis of messages between users and agents of a digital health service in Kenya
Source: Gates Open Res. 2019 May 29;3:1475. [Version 1] doi: 10.12688/gatesopenres.12999.1 (PMC6688461; doi:10.12688/gatesopenres.12999.1)
Supplement: Peer review history

# Peer review: Reviewer response for version 1

**DOI:** 10.21956/gatesopenres.14107.r27486

**Published:** 2019-08-08

**Authors:** Marleen Temmerman

**Affiliations:** 1 International Centre for Reproductive Health, Mombasa, Kenya

This is a well-written excellent paper, addressing an important new approach in the field of health education, more specifically sexual and reproductive health, not only using AI, but also two way communication transcript SMS methodology.

 The methodology is clearly established, sample size impressive and the results well presented, as well as the discussion section. References are adequate.

 A few remarks/questions:

1. How do the authors and the askNivi, differentiate between ‘Family Planning’ and ‘Contraceptives’ as both words are used for the same concept? Contraceptives are methods to prevent unplanned pregnancies, hence to ‘plan the family’.
2. For medical questions advice is asked from a nurse at a local maternity hospital. How does the project validate the correctness of the responses by the nurse? Some questions are difficult to address and the evidence is not always readily available. Is there a training/education/validation process set up to validate the responses?

Conclusion: positive advice for publication.

I confirm that I have read this submission and believe that I have an appropriate level of expertise to confirm that it is of an acceptable scientific standard.

# Peer review: Reviewer response for version 1

**DOI:** 10.21956/gatesopenres.14107.r27264

**Published:** 2019-06-04

**Authors:** Ona L. McCarthy

**Affiliations:** 1 Department of Population Health, Faculty of Epidemiology and Population Health, London School of Hygiene and Tropical Medicine, London, UK

This is a clear and concisely written report on a text mining analysis conducted on short messages sent via a SRH digital service ( *askNivi)*. The analysis included inbound and outbound messages sent from male and female user to and from *askNivi *agents. While I do not have experience in text mining, the techniques appear to be appropriate to answer the research question. I found the analysis fascinating. In addition, it is clear that AI is the way forward in the design of interactive and tailored digital interventions. The results (particularly the differences by gender and age) are useful for anyone planning to develop or analyse a similar service. One suggestion for additional information to include is to state how the random sample of 100 users selected for the conversation analysis.

I confirm that I have read this submission and believe that I have an appropriate level of expertise to confirm that it is of an acceptable scientific standard.
